# Changes in Time to Initial Physician Contact and Cancer Stage Distribution during the COVID-19 Pandemic in Patients with Head and Neck Squamous Cell Carcinoma at a Large Hungarian Cancer Center

**DOI:** 10.3390/cancers16142570

**Published:** 2024-07-18

**Authors:** Éva Szabó, Eszter Kopjár, László Rumi, Árpád Boronkai, Szabolcs Bellyei, Zoltán Gyöngyi, Antal Zemplényi, Balázs Sütő, János Girán, István Kiss, Éva Pozsgai, István Szanyi

**Affiliations:** 1 Department of Otorhinolaryngology, Clinical Center, University of Pécs, Munkácsy M. Street 2., 7621 Pécs, Hungary; 2 Urology Clinic, Clinical Center, University of Pécs, Munkácsy Mihaly Street 2, 7621 Pécs, Hungary; 3 Department of Oncotherapy, Clinical Center, University of Pécs, Édesanyák Street 17, 7624 Pécs, Hungary; 4 Department of Public Health Medicine, Medical School, University of Pécs, Szigeti Street 12, 7624 Pécs, Hungary; 5 Center for Health Technology Assessment and Pharmacoeconomics Research, Faculty of Pharmacy, University of Pécs, Rákóczi Street 2, 7623 Pécs, Hungary; 6 Skaggs School of Pharmacy and Pharmaceutical Sciences, University of Colorado, Anschutz Medical Campus, Aurora, CO 80045, USA; 7 Department of Anesthesiology and Intensive Therapy, Clinical Center, University of Pécs, Ifjúság Street 13, 7624 Pécs, Hungary; 8 Department of Primary Health Care, Medical School, University of Pécs, Rákóczi Street 2, 7623 Pécs, Hungary

**Keywords:** head and neck squamous cell carcinoma, COVID-19, cancer stage, time to initial physician contact, waiting time, symptom, predictive factor

## Abstract

**Simple Summary:**

Head and neck cancer is the seventh most common type of cancer worldwide, with high incidence and mortality rates, particularly in Hungary. The COVID-19 pandemic significantly impacted cancer care. Our study aimed to compare the characteristics and time to initial physician contact for patients with head and neck squamous cell carcinoma (HNSCC) before and during the pandemic at a large Hungarian cancer center. We conducted a retrospective study on patients aged 18 or older at Pécs Clinical Center, collecting demographic and clinical data from 1 January 2017, to 15 March 2020 (prepandemic), and from 16 March 2020, to 13 May 2021 (the pandemic period). The average number of monthly HNSCC diagnoses decreased by 12.4% during the pandemic. Early-stage cancers (I and II) increased, some advanced stages (III and IVa,c) decreased, and stage IVb cancers significantly rose. The median time from symptom onset to doctor visits increased from 43 to 61 days. This study highlights the pandemic’s impact on cancer diagnosis and care delays, revealing a bidirectional shift in cancer stages and emphasizing the need for more detailed analyses of COVID-19’s effects.

**Abstract:**

The aim of our study was to compare the characteristics and time to initial physician contact in patients with head and neck squamous cell carcinoma (HNSCC) before and during the COVID-19 pandemic at a large Hungarian cancer center. This was a retrospective study of patients 18 years or older presenting at the regional cancer center of Pécs Clinical Center with HNSCC between 1 January 2017, and 15 March 2020 (the pre-COVID-19 period) and between 16 March 2020, and 13 May 2021 (the COVID-19 period). Demographic and clinical data were collected, and the time between initial symptom onset and initial physician contact (TTP) was determined. Descriptive and exploratory statistical analyses were performed. On average, the number of patients diagnosed with HNSCC per month during the pandemic decreased by 12.4% compared with the pre-COVID-19 period. There was a significant increase in stage I and stage II cancers (from 15.9% to 20.3% and from 12.2% to 13.8%, respectively; p < 0.001); a decrease in stage III and IVa,c cancers; and a significant increase in stage IVb cancers (from 6% to 19.9%; p < 0.001) during the pandemic. The median TTP increased during the pandemic from 43 to 61 days (p = 0.032). To our knowledge, this is the first study investigating the effect of COVID-19 on patients with HNSCC in the Central–Eastern European region. We found a bidirectional shift in cancer stages and increased TTP during the pandemic. Our findings highlight the necessity for more nuanced analyses of the effects of COVID-19.

## 1. Introduction

Head and neck cancer is the seventh most common tumor type worldwide [1], with an increasing incidence rate [2] and a high mortality rate, particularly in Hungary, where it is among the top causes of cancer-related deaths [3]. In addition to the high number of premature deaths and related productivity loss [4], the treatment of head and neck cancer causes major direct medical costs [5,6].

Research has indicated that as the tumor size increases, the probability of controlling the cancer decreases, resulting in worse outcomes [7,8,9]. The risk for local cancer recurrence has been found to significantly increase with prolonged waiting times [7,10]. Therefore, organized efforts to decrease both patient-related and healthcare-related waiting times have become increasingly important [11].

A number of factors influence the waiting times for the diagnosis and treatment of head and neck cancer patients. Patient-related delays in contacting a physician for their symptoms may be due to socioeconomic conditions and psychopathological factors, or the accessibility of medical care [12,13,14]. Many head and neck cancer patients are primarily from lower social classes with risk factors like alcohol and tobacco use, who often delay seeking medical attention until severe symptoms, such as swallowing difficulties, occur [12,13]. Belonging to lower socioeconomic classes can limit access to medical care, especially for patients living in peripheral regions, due to the high costs of transportation and medical services [15]. Furthermore, research on cognitive psychology has indicated that the awareness and timing of symptom detection are influenced by a person’s inherent sensitivity to bodily changes and their unique situational factors [14]. Patient-related delays may have worsened during the COVID-19 pandemic, since tobacco use, alcohol consumption, and obesity rates were found to have significantly increased [8,16]. Additionally, global anxiety and depression rates rose by 25% [17], and higher unemployment rates, particularly affecting lower social classes, were linked to increased depression rates and suicidal behavior [17]. Delays in cancer care within healthcare systems can also stem from several factors, including inadequate diagnostic capacity, such as limited access to imaging techniques and insufficient numbers of trained professionals, which often result from constraints in both human and financial resources [18]. Another important factor may be the lack of clear-cut patient pathways. Health authorities in Western countries have developed policies and reforms for a more user-oriented healthcare service, including standardized pathways for specific patient groups like cancer patient pathways. Recent findings have indicated that these patient pathways can improve timely access to cancer diagnosis and treatment, reduce delays, and ultimately enhance patient outcomes [19,20,21]. 

The COVID-19 pandemic has had a significant impact on the care of oncological patients [22,23]. ENT specialists and dentists were particularly at risk of contracting and/or spreading the virus [16,24,25]. Based on the findings of international studies, delays in head and neck cancer care delivery and time to therapy were observed in many countries during the COVID-19 pandemic, which negatively affected tumor burden and overall survival. Studies reported that 22–43% of head and neck cancer cases were underdiagnosed during the pandemic, compared with the pre-COVID-19 period, mainly in elderly patients with oral cavity cancer [26,27]. In a Belgian study, a shift in the stages of laryngeal and oropharyngeal cancer was also observed in patients, resulting in an increased number of patients with advanced cancer at the time of diagnosis [28]. These investigations hold great significance in understanding the diagnosis and outcome of individuals with head and neck cancer, especially within the framework of unplanned events like the COVID-19 pandemic that may disrupt the functioning of healthcare systems. 

Hungary has the highest cancer incidence and mortality rates in Europe according to GLOBOCAN estimates [1]. Over the past decade, the incidence of the 10 most common types of cancer has increased in Hungary, while the number of cancer deaths has remained largely unchanged, resulting in approximately 33,000 deaths annually. Lung cancer, colorectal cancer, and breast cancer have the highest mortality rates, accounting for nearly half of all cancer deaths, while lip and oral cancer rank eighth in cancer mortality, causing 1311 deaths in 2019 [29]. 

To our knowledge, no Central European studies have been conducted to analyze the effects of the COVID-19 pandemic on the characteristics and healthcare-related waiting times of patients with head and neck cancer.

We hypothesized that in Hungary, as in several other countries, healthcare provision to cancer patients may have been delayed due to the increased burden of the COVID-19 pandemic on healthcare. Consequently, due to this heightened burden, along with lockdowns and restricted access to certain medical services, patients may not have sought medical advice in time, leading to the identification of more advanced-stage cancer patients during the pandemic.

Therefore, the aim of the present study was to analyze and compare the characteristics and time to initial physician contact of patients with head and neck squamous cell carcinoma (HNSCC) before and during the COVID-19 pandemic in a large Hungarian cancer center. It was also our objective to identify possible predictive factors of early- vs. advanced-stage cancer disease in the two study periods.

## 2. Methods

### 2.1. Setting

This study was carried out at a large Hungarian university clinic, the University of Pécs Clinical Center, the Department of Otorhinolaryngology and Head and Neck Surgery (UP ENT), located in Pécs, Hungary, with a dedicated cancer center (including an inpatient unit, a day oncology unit, and a radiotherapy unit) within the clinical center. As a regional center, the UP ENT is responsible for the treatment of cancer patients in Baranya County but also accepts patients from neighboring counties in the Transdanubian region of the country. Surgeries are performed on all head and neck regions from the thoracic inlet to the front and lateral skull base. In addition to primary tumor resection, neck dissections and reconstructive surgery are also performed. Alongside open surgery, transoral laser surgery and transoral robotic surgery are also part of the surgical activity. 

Our study received ethical approval from the Regional Ethical Committee prior to the research procedure (reference number: 8850-PTE2021).

### 2.2. Regulations Affecting the Hungarian Healthcare System during the COVID-19 Pandemic

The first, symptomatic COVID-19-positive patients in Hungary were documented at the beginning of March 2020. On 11 March 2020, the Hungarian government announced a national safety crisis, and 5 days later (16 March 2020), schools and preschools were closed, elective operations were rescheduled in healthcare centers across the country, and dentists were obligated to treat only emergency cases. Due to the low number of active COVID-19-positive cases, elective operations could be performed again within certain limits between May and November 2020; however, there was a state of pandemic preparedness. Throughout the COVID-19 pandemic period, there were no restrictions regarding oncological and emergency surgeries, cardiological interventions, and reproduction-related operations. On 13 May 2021, the national safety crisis ended, all restrictions were lifted, and the rules and regulations on the functioning of the healthcare system were returned to the prepandemic era. 

### 2.3. Study Design

This was an observational, retrospective study. The inclusion criteria were all patients 18 years or older, who visited the UP ENT between 1 January 2017 and 13 May 2021, with histological confirmation of squamous cell carcinoma of mucosal epithelial origin in the oral cavity, pharynx, and larynx, or cytology-confirmed cervical lymph node metastasis of an unknown primary tumor (CUP). The diagnostic criteria, therefore, meant that patients were included if they were given an International Classification of Diseases, 10th Revision (ICD-10) code of C00-C06, C09-C14, or C32. The exclusion criteria included the presence of any other tumor within 5 years prior to diagnosis and the presence of secondary tumors. 

The study interval was divided into two study periods, based on them being before or during the COVID-19 pandemic. The first period, between 1 January 2017 and 15 March 2020, was designated as the pre-COVID-19 period, and the second period, between 16 March 2020 and 13 May 2021, as the COVID-19 period. Determining the dates of the COVID-19 period was based on the regulations issued by the Hungarian National Directorate General for Hospitals regarding the changes in the provision of healthcare during the COVID-19 pandemic, as described above. 

Using automated data collection methods, the electronic database of the University of Pécs Clinical Center was screened for all patients who met the inclusion criteria, which included altogether 525 patients 18 years and older diagnosed with HNSCC in the full study interval. Collection of demographic data (sex distribution, the patient’s age at visit to the UP ENT, and place of residence) was carried out. Manual data collection was subsequently conducted to collect data on the types and frequency of main presentation symptoms (using the term described by the patient [30,31,32], the cancer type and stage, the specialty of the physician upon first contact, the dates of initial symptoms, the initial physician contact, the type of therapy, and death). The site of the tumor, chief complaints, and diagnoses given following UP ENT admission were classified according to the ICD-10. The date of initial symptoms was defined as the date given by the patient upon initial physician contact. The date of initial physician contact was the date of the patient first contacting any physician (a general practitioner, dentist, ENT specialist, or other specialist) with their symptoms. The time to initial physician contact (TTP) was the time between the appearance of initial symptoms to the initial physician contact.

The primary outcome measures for this study were the comparison of the clinical characteristics (symptoms, tumor site, and cancer stage) and median TTPs of HNSCC patients between the pre-COVID-19 and COVID-19 periods. The secondary outcome measures were the identification of possible predictive factors of early- vs. late-stage cancer disease in the two study periods. The investigated potential predictive factors included the main symptoms and tumor sites, as shown in Table 1.

The Strengthening the Reporting of Observational Studies in Epidemiology (STROBE) guidelines were used when designing and describing this study. 

Table 1 shows the demographic and clinical characteristics of HNSCC patients (n = 525) visiting the UP ENT in the two, pre-COVID-19 and COVID-19, study periods. 

Table 2 shows the time to initial physician contact (TTP) of HNSCC patients before and during the COVID-19 period. 

### 2.4. Data Analysis

The data analysis framework was developed to address the research questions set for this study. Both descriptive and exploratory approaches were used. Frequency tables were used to describe the demographic and clinical characteristics of the HNSCC cases and the number and time of death. The chi-square test was used to test the level of significance, which was considered significant at p < 0.05. Binary logistic regression analysis was conducted to identify factors that predicted contacting a physician (initial physician contact) in the early or late stages of the disease. The Mann–Whitney test was used to assess the significance level of the median values of the TTP. Statistical analyses were performed using the statistical software Jamovi 2.2.5.

## 3. Results

### 3.1. The Associations between Symptoms, Tumor Site, Cancer Stage, and Pre-COVID-19 and COVID-19 Study Periods

**Figure 1:**
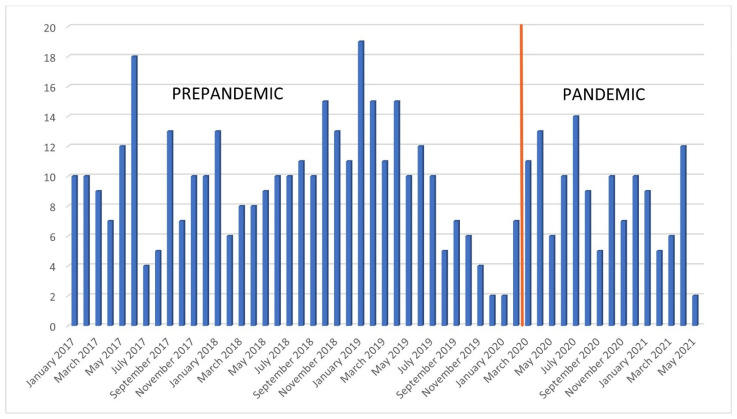
Number of patients per month presenting at the UP ENT before and during COVID-19 pandemic.

On average, the number of patients diagnosed with HNSCC per month during the pandemic decreased by 12.4% compared with the pre-COVID-19 period; however, this change was not statistically significant (Figure 1).

Since the four most common presenting symptoms were having a neck lump, pain, dysphagia, and an exulceration in the oral cavity, we analyzed their frequency between the two study periods, but no significant difference was found (Supplementary Table S1A–D). 

The relationship between the individual tumor sites (oral cavity, larynx, epipharynx, oropharynx, hypopharynx, and CUP) and the two study periods (Table 1) showed no significant association either, nor did we detect a significant difference in the distribution of early-stage (I–II) and advanced-stage (III–IV) cancers between the two study periods when categorizing them into these two groups (Table 1). 

To obtain a more nuanced analysis, we investigated the relationship between the individual cancer stages I-II-III-IVa,b,c and the two study periods and found a significant association. During the pandemic there was a significant increase in stage I and stage II cancers, a decrease in stage III and IVa,c cancers, and a significant increase in stage IVb cancers. The greatest rise was in IVb cancers, where the percentage of cancer pre-COVID-19 increased from 6.0% to 17.9% in the pandemic, while the greatest decrease was among stage IVa cancers (44.3% pre-COVID-19 and 33.3% during COVID-19). The proportion of patients with stage I cancer significantly increased from 15.9% to 20.3% during the COVID-19 period (Table 3).

### 3.2. Comparison of Waiting Time from Onset of Symptoms to Initial Physician Contact (TTP) of HNSCC Patients before and during the COVID-19 Period

When comparing the number of days from the appearance of first symptoms to initial physician contact (TTP), we found that the median TTP before the COVID-19 pandemic was significantly shorter than the median TTP during COVID-19 (43 vs. 61 days, respectively; p = 0.032), indicating that during the COVID-19 period, the median time to see a doctor increased by about 40% compared with the previous period (Table 4). The average number of days to initial physician contact was also longer during the pandemic than before it (Table 4). 

### 3.3. Predictive Factors of Tumor Stage in the Periods before and during the COVID-19 Pandemic

We investigated whether predictive factors of early-stage (stages I–II) and advanced-stage (stages III–IV) cancer could be identified among the examined parameters in this study. 

Through logistic regression analyses, we identified two cancer sites—oral cavity and laryngeal cancer—and two main symptoms—neck lump and dysphagia—which independently predicted increased odds of seeking medical attention at an early stage and advanced stage of the disease, respectively.

A laryngeal tumor and an oral cavity cancer in the pre-COVID-19 period increased the odds of visiting a physician at an early stage of cancer by 2.4 times and 3.3 times, respectively, compared with having a tumor in a different location. During the COVID-19 period, these odds significantly increased, with an OR of 4.57 for laryngeal cancer and 5.89 for oral cancer.

Thus, patients with HNSCC who had either laryngeal or oral cancer were more likely to contact a physician in an early stage of the disease during the pandemic than before the COVID-19 period (Table 5). 

Conversely, having a neck lump or dysphagia as a presenting symptom both before and during the COVID-19 period significantly increased the odds of patients visiting a physician in an advanced stage of cancer. 

Having a neck lump or dysphagia in the pre-COVID-19 period increased the odds of visiting a physician in an advanced stage of cancer by 7.4 times and 3.01 times, respectively, compared with having any other symptom. During the COVID-19 period, the odds significantly increased, with an OR of 4.97 for dysphagia, implying that those with dysphagia were more likely to contact a physician with an advanced-stage cancer during the pandemic than prior to it (Table 5).

## 4. Discussion

To our knowledge, our study is the first in this European region to investigate the differences in clinical characteristics, predictors, and time to initial physician contact in patients with HNSCC during the pre-COVID-19 and pandemic periods. Some of our findings align with previous investigations, such as an increased patient delay from symptom onset to initial physician contact during the pandemic. However, the bidirectional shift in tumor stages between the study periods presents a unique and unexpected effect of the pandemic.

The COVID-19 pandemic disrupted various aspects of healthcare. Due to lockdowns, personal physician–patient consultations decreased, nonurgent interventions were delayed, and a significant portion of healthcare resources was allocated to COVID-19 patients [33,34,35]. According to a Hungarian national cross-sectional study, there was a decrease in the number of visits to GPs and other specialists and a decrease in hospitalization rates, by 22.2%, 24.4%, and 6.7%, respectively, compared with the prepandemic period, while socioeconomic inequalities surrounding the provision of healthcare were also observed [34]. 

Cancer care was also affected, with over 55% of medical facilities reporting some degree of decreased provision of cancer care [8,36]. A decrease in the number of new HNSCC diagnoses during the COVID-19 pandemic has been reported, ranging from 7.5% to up to 50%, with 7.5%, 22%, 43%, and 50% reduction rates according to Japanese, US, Italian, and Canadian studies, respectively [10,26,27,37,38]. The decrease in the average number of newly diagnosed HNSCC patients per month also decreased during the pandemic in our study, by 12.4%, which is among the lower reduction rates compared with the previously mentioned studies. 

According to most investigations, the proportion of advanced-stage cancers increased during the pandemic, while the percentage of early-stage cancers decreased [26,27]. A significant increase in the overall proportion of stage T3/T4 cases [10,16,27], a significant increase in the T stage of squamous cell carcinoma [26,39], and a significant decrease in patients with early-stage cancer were observed during the pandemic compared with the pre-COVID-19 period [26,27,40], indicating that patients presented with more locoregionally advanced disease [39]. An explanation for this may have been that the delay in diagnosis allowed early-stage tumors to progress to advanced stages before patients sought medical care.

However, other analyses show different trends. For example, a population-based study in the Netherlands found no significant change in the proportion of advanced-stage HNSCC patients during the COVID-19 period. [28,40], and a large investigation of 4831 cases in Germany also observed no significant differences in tumor stage during the pandemic compared with the prepandemic era [41].

In line with these latter findings, we did not find any significant difference in the proportions of early- (I–II) and late-stage cancers (III–IV) during the pandemic in our study. When utilizing a more nuanced analysis, however, and investigating the four stages individually, including the IVa,b,c stages as well, we found that there was a more polarized distribution of the patients with certain cancer stages during the COVID-19 period, as opposed to before the pandemic, with a significant increase in early-stage I-II cancers, a decrease in stage III and IVa cancers, and a significant increase in IVb cancers, thus indicating a shift toward very early- and very late-stage cancers in the COVID-19 period. 

Waiting times have also been found to be affected by the pandemic, as reported in a Croatian study, which found a significant delay in initial presentation to an ENT specialist during the pandemic [36]. However, some studies have reported no significant changes in waiting times between the pre-COVID-19 and COVID-19 periods. Nishimura et al. found a 1.5-month median waiting time from the onset of subjective symptoms and the visit to a healthcare provider and Tevetoglu et al. reported a 3.4- and 2.9-week waiting period from first admission to surgery, with no statistically significant difference between the two study periods [26,39]. The median waiting times between symptom appearance and initial physician contact were comparable in our study (43 and 61 days pre-COVID-19 and during COVID-19); however, there was a significant increase in the TTP during the pandemic, indicating that patients waited longer with their symptoms to contact a physician. Shorter waiting times were reported in a Canadian investigation, however, with no significant difference in waiting times between the two periods (25 and 27 days prepandemic and during the pandemic, respectively) [27].

Some studies have reported a change in the types of symptoms and tumor sites within HNSCC due to the pandemic. According to recent studies, an increasing proportion of patients were diagnosed with oral cavity cancer compared with laryngeal cancer during the pandemic [16,36,42], possibly associated with increasing tobacco use, alcohol consumption, and obesity rates, which significantly rose during the pandemic [16,33,36,43]. In contrast with these findings, there is evidence that changes in symptomatology and tumor types were not observed in all countries [41], similar to our findings, where no significant change was found in the distribution of the tumor sites between the prepandemic and pandemic periods. Our results also show that there was no significant relationship between the four most common symptoms (neck lump, dysphagia, oral exulceration, and pain) and the two study periods. We did find, however, that laryngeal and oral cavity cancer were predictors of early-stage cancer, while having a neck lump and dysphagia were predictive of advanced-stage cancer, and that the odds of these parameters for predicting early- or advanced-stage cancer changed during the pandemic. Our results indicate that patients with dysphagia waited longer to contact a physician than patients with other symptoms during the COVID-19 period than before. Meanwhile, patients with laryngeal and oral cavity cancer were more likely to contact a physician with their symptoms in an early stage of their cancer during the pandemic than before. One possible reason for earlier physician contact and diagnosis of laryngeal tumors might be that HNSCC patients consider hoarseness a concerning symptom, prompting them to seek medical help. Likewise, oral cavity cancer, characterized by painful oral ulcers and noticeable changes in the oral mucosa, may encourage patients to seek medical attention promptly [44]. 

Dysphagia, a feeling of unpleasant swallowing, is often associated with a sore throat, which can be a symptom of an upper respiratory infection [45]. Thus, it is possible that some patients during the pandemic might have mistaken their symptoms for COVID-19 infection. This could have led to delayed initial visits to physicians, as many patients with mild upper respiratory symptoms were advised to stay home when contacting their GP via telephone. Furthermore, it is likely that when patients contacted a physician, typically their family doctor, about pain from oral ulceration, they may have been advised to stay home due to the possibility that this symptom could indicate stomatitis. This advice could have contributed to delays in seeking further medical evaluation and treatment.

Previous studies indicate the pandemic’s negative impact on HNSCC cancer stage progression [28,37], but recent evidence challenges the idea of a universal stage shift. Our study reveals a unique pattern: instead of a uniform progression to later stages, we observed a polarization, with significantly higher proportions of stage I and IVb cases, thus indicating a dual effect. Some individuals may have delayed seeking medical attention due to lockdowns and restricted access to certain forms of medical care, unawareness about cancer symptoms, or downplaying their symptoms, such as pain. Conversely, others may have been more vigilant about their symptoms, possibly influenced by heightened awareness of COVID-19 symptoms. This latter phenomenon may possibly be due to the similarity between upper respiratory symptoms of COVID-19 and those of head and neck cancers, coupled with extensive media coverage emphasizing COVID-19 symptom awareness during the pandemic, heightened public recognition of these symptoms, and increased medical consultations. This hypothesis finds support in prior studies, including recent research using large-scale data that examined behavioral changes in response to the pandemic and revealed that the WHO’s pandemic declaration significantly increased public awareness, with a notable surge in searches for COVID-19 information and interest in testing, ultimately aiding in case identification [46]. Furthermore, previous publications have also highlighted that awareness regarding cancer plays a crucial role in promoting behaviors that facilitate early detection of cancer. [47] In short, as an indirect consequence of the pandemic, increased awareness of symptoms may have unintentionally prompted more patients to seek medical care earlier than they would have before the pandemic. Our additional findings further support this theory, indicating a rise in early-stage cancers and a greater tendency for early physician consultation among patients with oral cavity and laryngeal cancers during the pandemic. Conversely, delays in seeking medical care, indicated by a longer median TTP, and later initial physician contact with dysphagia were also noted. 

Low awareness levels highlight the necessity for health education and sensitization regarding cancer and its various aspects [47]. Increased media coverage during events like pandemics enhances public awareness of diseases, including cancer symptoms [46]. Therefore, promoting awareness about cancer symptoms should remain a priority even in non-pandemic times.

Further investigations into the attitudes of HNSCC patients toward COVID-19 and other diseases, as well as changes in the healthcare system, are needed to provide valuable insights into patient behaviors.

Our study’s limitations include its single-center design, potentially limiting its generalizability nationwide. Additionally, the accuracy of the data on the time from symptom onset to initial physician contact may be compromised due to their subjective nature, varying significantly among patients.

## 5. Conclusions

In summary, our study revealed a bidirectional shift in cancer stages, marked by a significant increase in both very early-stage (stage I) and very advanced-stage (stage IVb) cancers, while the distribution of early-stage (I–II) and advanced-stage (III–IV) cancers showed no significant difference. Additionally, we observed a notable increase in the time to initial physician contact during the COVID-19 pandemic. Patients with dysphagia experienced prolonged delays in contacting a physician compared with before the pandemic, whereas patients with oral cavity or laryngeal cancer were more inclined to seek medical attention in the early stages of their cancer during the pandemic compared with the prior period.

Our findings emphasize the importance of conducting nuanced analyses to understand the varied impacts of COVID-19. Such insights are essential not only for mitigating the adverse effects of potential future health crises on HNSCC patients but also for promoting awareness and encouraging timely healthcare contact when symptoms arise.

## Figures and Tables

**Table 1:** The demographic and clinical characteristics of HNSCC patients visiting the UP ENT before and during the COVID-19 period.

	Total n = 525 (%)	Before COVID-19 n = 402 (%)	During COVID-19 n = 123 (%)	p
**Total number**	525	402	123	
**Sex**				0.434
**Male**	427 (81.3)	324 (80.6)	103 (83.7)	
**Female**	98 (18.7)	78 (19.4)	20 (16.3)	
**Age group (years)**				0.708
**18–54**	78 (14.8)	59 (14.7)	19 (15.4)	
**55–59**	88 (16.7)	68 (16.9)	20 (16.3)	
**60–64**	107 (20.4)	82 (20.4)	25 (20.3)	
**65–69**	117 (22.3)	88 (21.9)	29 (23.6)	
**70–74**	63 (12)	45 (11.2)	18 (14.6)	
**>75**	72 (13.7)	60 (14.9)	12 (9.8)	
**Place of residence**				0.203
**County seat**	140 (26.7)	109 (27.1)	31 (25.2)	
**Other city**	175 (33.3)	126 (31.3)	49 (39.8)	
**Village**	210 (40)	167 (41.5)	43 (35)	
**Tumor site**				0.507
**Lips and oral cavity**	152 (28.9)	112 (27.9)	40 (32.5)	
**Oropharynx**	137 (26.1)	105 (26.1)	32 (26)	
**Hypopharynx**	93 (17.8)	70 (17.4)	23 (18.7)	
**Larynx**	120 (22.9)	94 (23.4)	26 (21.1)	
**Epipharynx**	8 (1.5)	8 (2)	0(0)	
**CUP (carcinoma of unknown primary)**	15 (2.9)	13 (3.2)	2 (1.6)	
**Frequency of the main symptoms**				0.076
**1**	230 (43.9)	166 (41.3)	64 (52.5)	
**2**	181 (34.5)	143 (35.6)	38 (31.1)	
**3 or more**	113 (21.5)	93 (23.1)	20 (16.4)	
**Specialty of initially contacted physician**				0.441
**ENT specialist**	384 (73.4)	291 (72.4)	93 (75.6)	
**Dentist**	112 (21.3)	86 (21.4)	26 (21.1)	
**Other**	29 (5.5)	25 (6.2)	4 (3.3)	
**Tumor stage**				0.199
**Early stage (I–II)**	155 (29.5)	113 (28.1)	42 (34.1)	
**Late stage (III–IV)**	370 (70.5)	289 (71.9)	81 (65.9)	

**Table 2:** The time to initial physician contact (TTP) * in HNSCC patients before and during the COVID-19 period.

	TTP in Days					p
	0–30	31–90	91–180	181–359	360–	
**Before COVID-19 n = 401 (%)**	131 (32.9)	167 (41.6)	63 (15.7)	22 (5.5)	17 (4.2)	0.292
**During COVID-19** **n = 122 (%)**	29 (23.8)	52 (42.6)	26 (21.3)	9 (7.4)	6 (4.9)	
**Total n = 523 (%)**	161 (30.8)	219 (41.9)	89 (17)	31 (5.9)	23 (4.4)	

* TTP: time between the appearance of initial symptoms to the initial physician contact.

**Table 3:** The association between tumor stage and the two study periods.

	Stage I	Stage II	Stage III	Stage IVa	Stage IVb	Stage IVc	p
**Before COVID-19 n = 402 (%)**	64 (15.9)	49 (12.2)	68 (16.9)	178 (44.3)	24 (6)	19 (4.7)	**<0.001**
**During COVID-19** **n = 123 (%)**	25 (20.3)	17 (13.8)	13 (10.6)	41 (33.3)	22 (17.9)	5 (4.1)	
**Total n = 525 (%)**	89 (17)	66 (12.6)	81 (15.4)	219 (41.7)	46 (8.8)	24 (4.6)	

**Table 4:** Median time to initial physician contact (TTP) in HNSCC patients before and during the COVID-19 period.

		Before COVID-19 n = 401	During COVID-19 n = 122	p
**TTP in days, range (0–732)**	**Mean** **Median**	77.67 43	83.82 61	**0.032**

**Table 5:** Predictive factors of early-stage and late-stage HNSCC in the periods before and during the COVID-19 pandemic.

		Before COVID-19 OR	During COVID-19 OR
**Early stage**	Larynx	2.41 (1.306–4.442)	4.57 (1.601–13.057)
	Oral cavity	3.31 (1.850–5.907)	5.89 (2.292–15.158)
**Advanced stage**	Neck lump	7.04 (3.236–15.299)	3.01 (1.572–5.760)
	Dysphagia	6.93 (1.932–24.861)	4.97 (1.351–18.250)

## Data Availability

The datasets used and/or analyzed during the current study are available from the corresponding author on reasonable request.

## Supplementary Materials

The following supporting information can be downloaded at: https://www.mdpi.com/article/10.3390/cancers16142570/s1, Table S1: The relationship between the four most common symptoms (neck lump, dysphagia, oral exulceration, and pain) and the two study periods.

## Abbreviations

**Abbreviation**	**Full Name**
HNSCC	Head and neck squamous cell carcinoma
UP CC ENT	University of Pécs Clinical Centre, the Department of Otorhinolaryngology
TTP	Time to initial physician contact
CUP	Carcinoma of unknown primary
