# Supplementary material for: Changes in Time to Initial Physician Contact and Cancer Stage Distribution during the COVID-19 Pandemic in Patients with Head and Neck Squamous Cell Carcinoma at a Large Hungarian Cancer Center
Source: Cancers (Basel). 2024 Jul 18;16(14):2570. doi: 10.3390/cancers16142570 (PMC11274479; doi:10.3390/cancers16142570)
Supplement: Supplementary file 1 [file cancers-16-02570-s001.zip › cancers-3067624-supplementary.pdf]

SUPPLEMENTARY MATERIAL: The relationship between the four most common symptoms A) *neck lump, B) dysphagia,* C) *oral exulceration* and the two study periods.

Supplementary Table S1A

	**Neck lump**	**Other**	**p**
**Before Covid-19 n=402 (%)**	110 (27.4)	292 (72.6)	
**During Covid-19 n=123 (%)**	30 (24.4)	93 (75.6)	0.514
**Total n=525 (%)**	140 (26.7)	385 (73.3)	

Supplementary Table S1B

	**Pain**	**Other**	**p**
**Before Covid-19 n=402 (%)**	103 (25.6)	299 (74.4)	
**During Covid-19 n=123 (%)**	31 (25.2)	92 (74.8)	0.926
**Total n=525 (%)**	134 (25.5)	391 (74.5)	

Supplementary Table S1C

	**Dysphagia**	**Other**	**p**
**Before Covid-19 n=402 (%)**	99 (24.6)	303 (75.4)	
**During Covid-19 n=123 (%)**	24 (19.5)	99 (80.5)	0.241
**Total n=525 (%)**	123 (23.4)	402 (76.6)	

Supplementary Table S1D

	**Exulceration in the oral cavity**	**Other**	**p**
**Before Covid-19 n=402 (%)**	85 (21.1)	317 (78.9)	
**During Covid-19 n=123 (%)**	32 (26%)	91 (74)	0.256
**Total n=525 (%)**	117 (22.3)	408 (77.7)	
